# Surgical treatment of long head of biceps pathology: analyzing trends in the United States from 2010 to 2019

**DOI:** 10.1016/j.xrrt.2024.12.013

**Published:** 2025-01-23

**Authors:** Jacob A. Worden, John M. Kopriva, Henry M. Gass, Zaamin B. Hussain, Anthony L. Karzon, Krishna N. Chopra, Michael B. Gottschalk, Eric R. Wagner

**Affiliations:** a Department of Orthopaedic Surgery, Medical College of Georgia, Augusta, GA, USA; b Department of Orthopaedic Surgery, Emory University School of Medicine, Atlanta, GA, USA

**Keywords:** Arthroscopic tenodesis, Biceps pathology, Superior labrum, Tenodesis, Tenotomy, Trends analysis

## Abstract

**Background:**

The long head of the biceps tendon (LHBT) is a common cause of anterior shoulder pain. A symptomatic LHBT is commonly encountered in the setting of a rotator cuff tear. The purpose of this study was to determine trends in the incidence of isolated tenotomy and tenodesis procedures for symptomatic LHBT in the setting of rotator cuff repairs (RCR).

**Methods:**

The MarketScan database was queried from 2010 to 2019 for biceps tenotomy (open) and tenodesis (open and arthroscopic) in the United States. Annual procedure volumes and incidences were calculated using discharge weights and U.S. Census Bureau data, respectively. Further subgroup analysis included age, gender, and region.

**Results:**

Open tenodesis remained the most common procedure in the United States for isolated LHBT management. Its incidence increased by 180% from 2010 to 2019, accounting for 49% of isolated LHBT procedures by 2019. In the setting of RCR, arthroscopic tenodesis was most common, and its incidence grew by 138%. By 2019, arthroscopic tenodesis accounted for 58% of procedures in the setting of RCR, while tenotomy claimed only 2%. The incidence of all procedures increased for the age ≥65 cohort, with a notable 828% increase in the incidence of open tenodesis as an isolated procedure, accounting for 76% of procedures by 2019.

**Conclusion:**

Volumes of procedures aimed to ameliorate LHBT pathology increased from 2010 to 2019. Open tenodesis remained the preferred procedure for isolated LHBT pathology, while arthroscopic tenodesis was preferred in the setting of concomitant RCR. Future research can develop algorithmic approaches to treating biceps pathology.

Long head of the biceps tendon (LHBT) pathology is often responsible for anterior shoulder pain.^4^ Although patients may suffer from an isolated tendinopathy, it is commonly associated with rotator cuff and labral pathology.^4^^,^^19^ Operative management may be warranted in cases where conservative measures, such as therapy and injections, fail to ameliorate symptoms. Biceps tenodesis and tenotomy are two commonly used surgical interventions for this pathology, without significant differences in functional outcomes and patient satisfaction.^1^^,^^2^^,^^17^^,^^20^^,^^28^ Proponents of tenotomy cite shorter operative times, decreased cost, and faster recovery,^27^ whereas surgeons who prefer tenodesis note increased strength and resistance to load, less muscle cramping, and better cosmesis by avoiding the “Popeye” deformity.^12^^,^^17^^,^^25^^,^^28^ For tenodesis, arthroscopic techniques have advanced in recent decades, potentially reducing the risk of surgical site infection and nerve injury by avoiding incisions around the axilla.^1^^,^^13^^,^^14^^,^^22^ In direct comparisons between open and arthroscopic LHBT tenodesis, however, no substantial clinical differences have been noted.^1^^,^^13^^,^^15^

Numerous studies have investigated trends in isolated LHBT tenodesis,^3^^,^^21^^,^^23^^,^^24^ isolated biceps tenotomy,^3^ and isolated rotator cuff repairs (RCR).^5^^,^^8^ These studies, however, use older data that precede the emergence of recently developed novel arthroscopic techniques and comparison studies.^1^^,^^13^^,^^15^^,^^17^^,^^20^ Additionally, trends in the utilization of biceps tenodesis vs. tenotomy in isolation or with concomitant RCR have not been well reported. Finally, it has yet to be proven whether tenodesis or tenotomy is superior.^11^^,^^27^ Analyzing the latest procedural trends may help us better understand standards of practice across the country, serving as a foundation for future research to develop an algorithmic approach to treat LHBT pathology in isolation or in the setting of rotator cuff pathology.

Our study sought to determine the national trends in open and arthroscopic LHBT tenodesis and open LHBT tenotomy, both in isolation and in conjunction with RCR. We hypothesized that, given low complication rates and high patient satisfaction, the incidence of biceps tenotomy, open tenodesis, and arthroscopic tenodesis has all increased over the past decade (2010-2019), both in isolation and with RCR.

## Methods

This retrospective cohort study utilized the IBM MarketScan Commercial Claims and Encounters and Medicare Supplemental databases. These databases are among the largest administrative claims databases assimilated from insurance claims of over 300 employer-sponsored health plans and Medicare supplemental plans. Over 245 million unique patients are included, and records include inpatient and outpatient encounters, surgical procedures, and pharmaceutical prescriptions. These national insurance databases were used in conjunction with population estimates from the U.S. Census Bureau to estimate the annual incidence and procedural trends of open biceps tenodesis, arthroscopic biceps tenodesis, and biceps tenotomies in isolation and in conjunction with RCR from 2010 to 2019. As a review of publicly available, deidentified data from the IBM MarketScan Research Database, this study was exempt from institutional review board approval.

Current Procedural Terminology (CPT) codes 23405 (tenotomy, shoulder area; single tendon), 23430 (tenodesis of long head of biceps), and 29828 (arthroscopy, shoulder, surgical; biceps tenodesis) were used to search MarketScan for all primary biceps tenodesis and tenotomy procedures. Patients were excluded if they underwent a total shoulder arthroplasty. Patients who underwent RCR were excluded if they did not have their RCR on the same date as their biceps tenodesis or tenotomy. Demographic information (age, gender, and geographic region of procedure) was collected for all patients.

Statistical analysis was performed using SAS, version 9.4 (SAS Institute). IBM MarketScan discharge weights, constructed from Public Use Microdata Sample of the American Community Survey, were used to help determine national estimates of the procedures with the Complex Samples function of the IBM SPSS Statistics software (IBM Corp., Armonk, NY, USA). IBM MarketScan provides completion factors to determine the number of months of data for healthcare services and an annualization factor for rate calculations. These can be used to derive weighted population averages. These weights were incorporated when calculating incidence using the appropriate statistical software functions. This allowed national incidence to be calculated and stratified using the appropriate population numbers of the respective year and group (annual total, annual male/female, annual geographical region) published by the U.S. Census Bureau. Population estimates from the U.S. Census Bureau were used in conjunction with the IBM MarketScan database to estimate the annual volume and incidence of the procedures in the United States. Volume and incidence were then estimated for gender and age subgroups and four geographical regions of the United States to include the Northeast, Midwest, South, and West. National estimates were determined with 95% confidence intervals. An absence of confidence interval overlapping between incidence values of both procedural groups demonstrated statistical significance.

## Results

### Trends in isolated tenotomy and tenodesis

**Figure 1:**
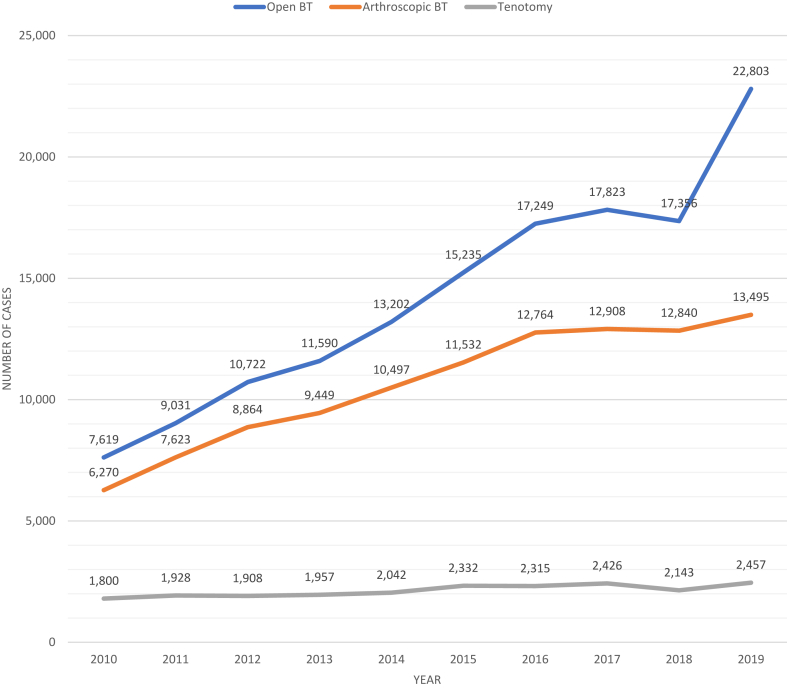
Case volumes: open vs. arthroscopic isolated biceps tenodesis vs. isolated biceps tenotomy. *BT*, biceps tenodesis.

**Figure 2:**
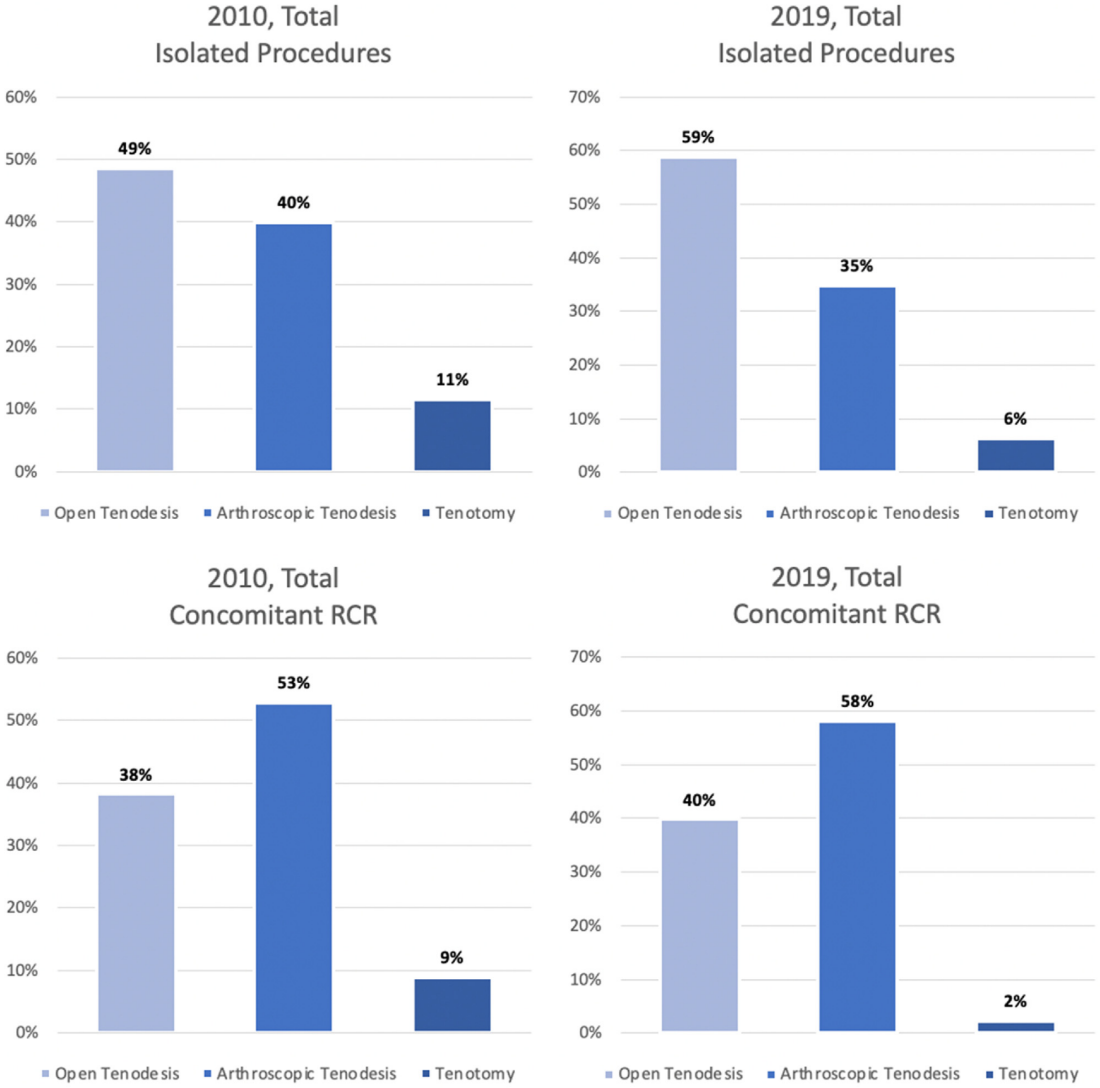
Procedure volumes over time: stratification of individual procedure volumes as a percentage of overall procedure volume. *RCR*, rotator cuff repair.

An estimated total of 149,488 isolated open biceps tenodesis procedures and 111,540 isolated arthroscopic biceps tenodesis procedures took place over the 10-year period from 2010 to 2019, while only 23,013 isolated biceps tenotomies occurred (Table I). All three procedures trended positively over the study period (Fig. 1). For isolated LHBT pathology, the incidence of open tenodesis increased by 180%, and arthroscopic tenodesis increased by 101%, whereas tenotomy increased by only 28% (Table II). In 2010, isolated LHBT procedures consisted of 49% open tenodesis, 40% arthroscopic tenodesis, and 11% tenotomy (Fig. 2). By 2019, isolated LHBT procedures consisted of 59% open tenodesis, 35% arthroscopic tenodesis, and 6% tenotomy.

**Table I:** Total procedure volumes from 2010 to 2019.

Total isolated procedure volumes	Total procedure volumes with concomitant RCR
Open tenodesis	Open tenodesis + RCR
149,488 (147,394-151,582)	209,951 (207,432-212,471)
Arthroscopic tenodesis	Arthroscopic tenodesis + RCR
111,540 (109,796-113,283)	299,917 (297,007-302,828)
Tenotomy	Tenotomy + RCR
23,013 (22,147-23,879)	30,704 (29,630-31,778)
Total	Total
284,040 (281,374-286,707)	540,573 (537,067-544,078)

*RCR*, rotator cuff repairs.

Variables represented as estimates with 95% confidence intervals.

**Table II:** Volume and incidence estimates by year: open vs. arthroscopic isolated biceps tenodesis vs. isolated biceps tenotomy.

Y	Open tenodesis		Arthroscopic tenodesis		Tenotomy	
	Volume	Incidence	Volume	Incidence	Volume	Incidence
2010	7619 (7284-7953)	25.1 (23.96-26.16)	6270 (5969-6571)	20.6 (19.64-21.62)	1800 (1626-1974)	5.9 (5.35-6.50)
2011	9031 (8657-9405)	29.5 (28.24-30.68)	7623 (7259-7988)	24.9 (23.67-26.05)	1928 (1762-2094)	6.3 (5.75-6.83)
2012	10,722 (10,333-11,111)	34.7 (33.43-35.94)	8864 (8490-9238)	28.7 (27.46- 29.88)	1908 (1727-2090)	6.2 (5.59-6.76)
2013	11,590 (11,149-12,0301)	37.2 (35.79-38.62)	9449 (9028-9869)	30.3 (28.99-31.68)	1957 (1772-2143)	6.3 (5.69-6.88)
2014	13,202 (12,722-13,681)	42.0 (40.50-43.55)	10,497 (10,051-10,943)	33.4 (32.00-34.84)	2042 (1841-2244)	6.5 (5.86-7.14)
2015	15,235 (14,414-16,057)	48.1 (45.54-50.73)	11,532 (10,758-12,307)	36.4 (33.99-38.88)	2332 (1922-2741)	7.4 (6.07-8.66)
2016	17,249 (16,567-17,928)	54.1 (52.02-56.28)	12,764 (12,183-13,345)	40.1 (38.24-41.89)	2315 (2060-2571)	7.3 (6.47-8.07)
2017	17,823 (17,061-18,584)	55.5 (53.15-57.89)	12,908 (12,258-13,557)	40.2 (38.19-42.23)	2426 (2155-2696)	7.6 (6.71-8.40)
2018	17,356 (16,584-18,127)	53.7 (51.36-56.14)	12,840 (12,204-13,476)	39.8 (37.79-41.73)	2143 (1886-2400)	6.6 (5.84-7.43)
2019	22,803 (21,531-24,075)	70.2 (66.31-74.15)	13,495 (12,644-14,347)	41.6 (38.94-44.19)	2457 (2013-2900)	7.6 (6.20-8.93)
% change	199%	180%	115%	101%	37%	28%

Incidence is reported per 1,000,000; % change represents the difference from 2010 to 2019.

Variables represented as estimates with 95% confidence intervals.

**Figure 3:**
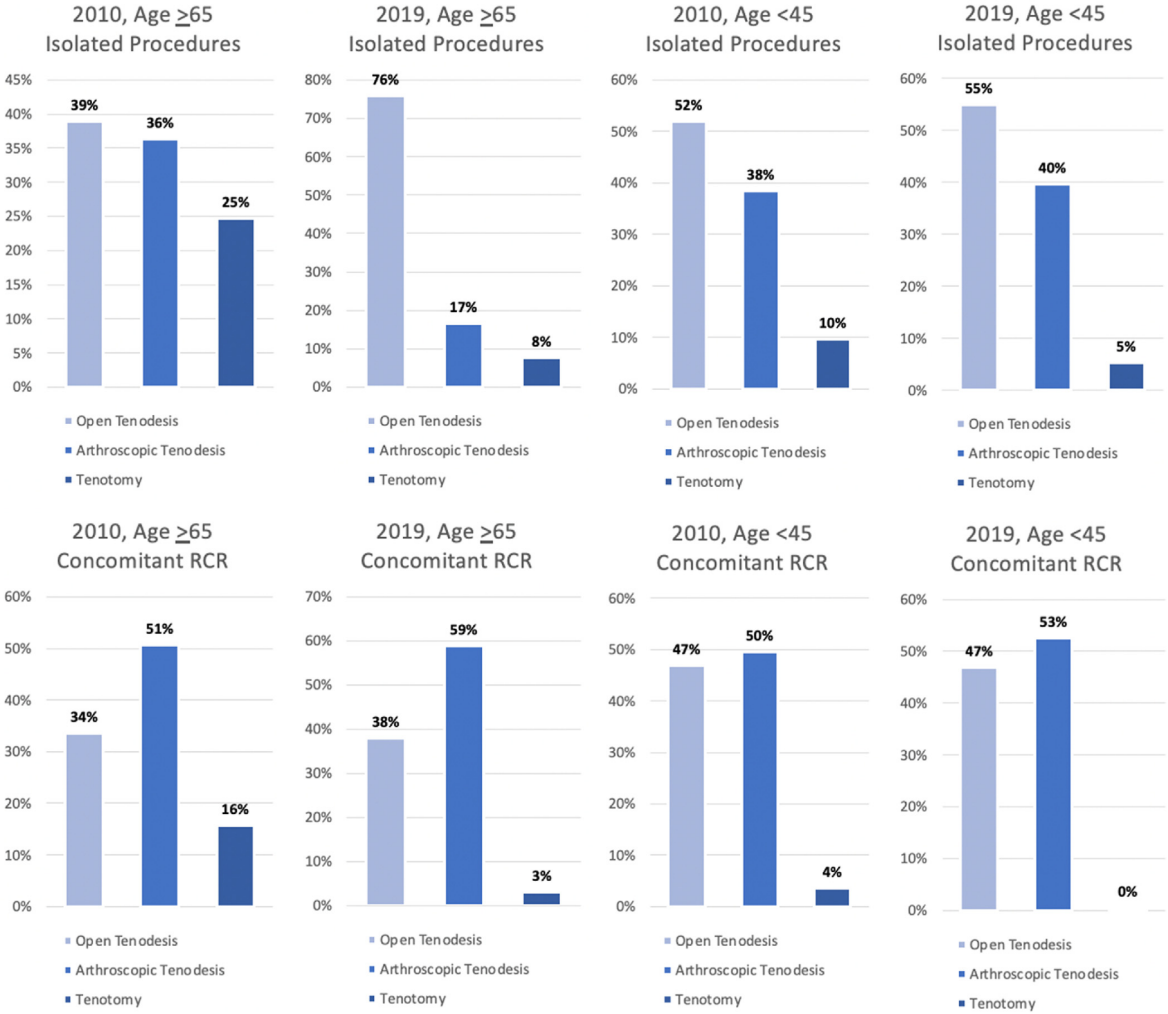
Procedure volumes by age: stratification of individual procedure volumes as a percentage of overall procedure volume. *RCR*, rotator cuff repair.

Trends in isolated procedures were then investigated between four age groups (age <45 years, 45-54 years, 55-64 years, and >65 years) (Table III). From 2010 to 2019, both open and arthroscopic tenodesis saw increases in volume and incidence throughout every age group. Specifically, the age <45 years cohort had increased incidences of open and arthroscopic tenodesis of 173% and 167%, respectively. The distribution of isolated LHBT procedures among the age <45 cohort in 2019 compared to 2010 (Fig. 3) reflects the increase in tenodesis procedures relative to tenotomy, with a 2019 procedure distribution of 55% open tenodesis, 40% arthroscopic tenodesis, and only 5% tenotomy.

**Table III:** Volume and incidence by age group: open vs. arthroscopic isolated biceps tenodesis vs. isolated biceps tenotomy.

	Volume			Incidence		
	2010	2019	% change	2010	2019	% change
Open tenodesis						
<45 yrs	2208 (2034-2382)	6130 (5650-6610)	177.6∗	11.8 (10.90-12.76)	32.2 (29.72-34.77)	172.6∗
45-54 yrs	2797 (2608-2985)	5574 (5136-6013)	99.3∗	63.1 (58.88-67.38)	132.5 (122.07-142.91)	109.9∗
55-64 yrs	2194 (2022-2366)	5993 (5543-6443)	173.1∗	64.0 (59.00-69.01)	143.5 (132.74-154.29)	124.2∗
≥65 yrs	420 (288-552)	5107 (4106-6108)	1116.4∗	10.8 (7.42-14.25)	100.6 (80.84-120.26)	828.2∗
Arthroscopic tenodesis						
<45 yrs	1629 (1483-1776)	4422 (4004-4840)	171.4∗	8.70 (7.95-9.51)	23.30 (21.07-25.46)	166.5∗
45-54 yrs	2277 (2107-2446)	4150 (3775-4526)	82.3∗	51.40 (47.56-55.22)	98.70 (89.72-107.58)	92.0∗
55-64 yrs	1973 (1814-2132)	3806 (3462-4149)	92.9∗	57.6 (52.92-62.19)	91.1 (82.91-99.36)	58.3∗
≥65 yrs	391 (266-516)	1117 (574-1660)	185.6∗	10.1 (6.86-13.33)	22.0 (11.31-32.69)	117.9
Tenotomy						
<45 yrs	409 (332-485)	592 (446-737)	44.8	2.2 (1.78-2.60)	3.1 (2.35-3.88)	42.2
45-54 yrs	512 (431-593)	639 (483-794)	24.8	11.6 (9.73-13.38)	15.2 (11.49-18.88)	31.4
55-64 yrs	613 (522-704)	710 (551-870)	15.9	17.9 (15.24-20.53)	17.0 (13.19-20.84)	−4.9
≥65 yrs	266 (167-365)	516 (161-870)	93.6	6.9 (4.32-9.43)	10.2 (3.18-17.14)	47.7

Incidence reported per 1,000,000.

Variables represented as estimates with 95% confidence intervals.

∗ Represents a statistically significant change.

The age >65 years cohort underwent more dramatic changes in procedure distribution over the study period (Fig. 3). In 2010, there was relative balance of isolated LHBT procedures with 39% open tenodesis, 36% arthroscopic tenodesis, and 25% tenotomy. Over the study period, the incidence of open tenodesis among the age >65 cohort increased by 828%. By 2019, the distribution of isolated procedures was 76% open tenodesis, 17% arthroscopic tenodesis, and 8% tenotomy.

**Figure 4:**
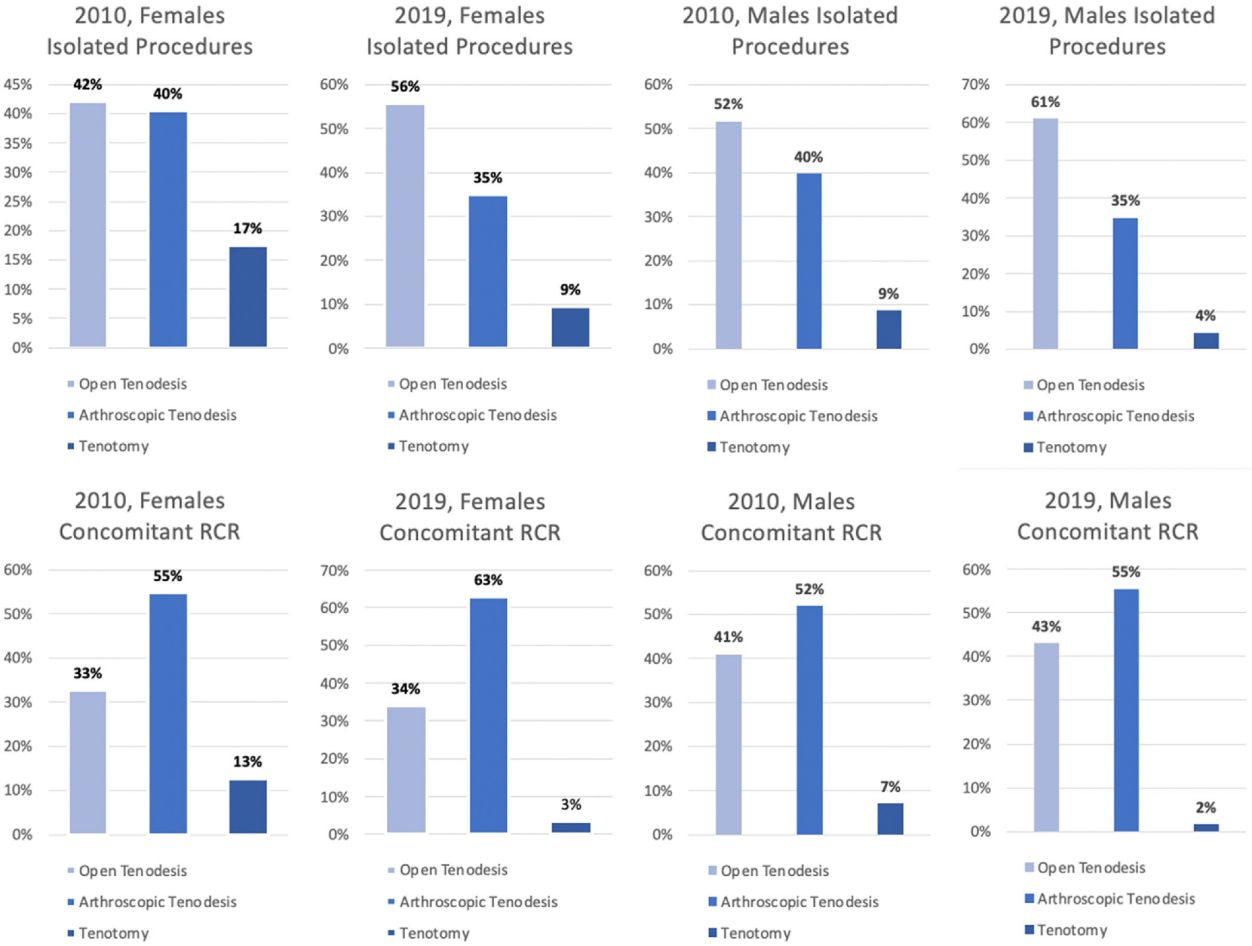
Procedure volumes by gender: stratification of individual procedure volumes as a percentage of overall procedure volume. *RCR*, rotator cuff repair.

When comparing trends in isolated procedures between genders, both males and females had significant increases in open and arthroscopic tenodesis (Table IV). As an isolated procedure, open tenodesis remained the most common for both genders and increased in proportion relative to arthroscopic tenodesis and tenotomy (Fig. 4). Although the volume and incidence of tenotomy statistically increased for females, the proportion of isolated procedures decreased in comparison to open and arthroscopic tenodesis.

**Table IV:** Volume and incidence by gender: open vs. arthroscopic isolated biceps tenodesis vs. isolated biceps tenotomy.

	Volume			Incidence		
	2010	2019	% change	2010	2019	% change
Open tenodesis						
Male	5494 (5207-5780)	14,410 (13,457-15,364)	162.3∗	37 (34.85-38.69)	90.1 (84.17-96.09)	145.1∗
Female	2125 (1951-2299)	8393 (7547-9238)	295.0∗	14 (13.7-12.62)	50.9 (45.79-56.05)	270.4∗
Arthroscopic tenodesis						
Male	4228 (3975-4481)	8214 (7543-8886)	94.3∗	28 (26.60-30.00)	51.4 (47.18-55.58)	81.5∗
Female	2042 (1878-2205)	5281 (4755-5807)	158.6∗	13 (12.15-14.27)	32.0 (28.85-35.23)	142.6∗
Tenotomy						
Male	918 (786-1050)	1028 (695-1362)	12.0	6.1 (5.26-7.03)	6.4 (4.35-8.52)	4.7
Female	882 (769-995)	1428 (1137-1720)	61.9∗	5.7 (4.97-6.44)	8.7 (6.90-10.44)	51.8∗

Incidence reported per 1,000,000.

Variables represented as estimates with 95% confidence intervals.

∗ Represents a statistically significant change.

Geographic stratification also showed increases in both open and arthroscopic tenodesis for isolated LHBT procedures (Table V). Regarding the distribution of the three procedures in 2010 vs. 2019, all four regions (Northeast, Midwest, South, and West) saw similar increases in the proportion of open tenodesis and similar decreases in the proportion of tenotomy. The incidence of tenotomy did significantly increase in the West but remained only 9% of total isolated procedures for LHBT in that region (with open tenodesis making up 60% of procedures, for reference).

**Table V:** Volume and incidence by region: open vs. arthroscopic isolated biceps tenodesis vs. isolated biceps tenotomy.

	Volume			Incidence		
	2010	2019	% change	2010	2019	% change
Open tenodesis						
Northeast	1283 (1140-1425)	3039 (2539-3647)	136.9∗	23.6 (21.00-26.25)	54.8 (45.80-65.78)	136.1∗
Midwest	2101 (1908-2293)	5827 (5355-6298)	177.3∗	32.0 (29.06-34.91)	86.6 (79.55-93.55)	170.6∗
South	2412 (2241-2582)	8580 (7769-9392)	255.7∗	21.8 (20.27-23.35)	70.3 (63.70-77.00)	222.6∗
West	1771 (1610-1932)	5303 (4640-5967)	199.4∗	25.5 (23.17-27.80)	69.5 (60.84-78.24)	172.9∗
Arthroscopic tenodesis						
Northeast	1159 (1022-1297)	1484 (1252-1717)	28.0	21.3 (18.82-23.88)	26.8 (22.58-30.97)	25.4
Midwest	1669 (1498-1840)	3245 (2891-3599)	94.4∗	25.4 (22.81-28.02)	48.2 (42.94-53.46)	89.7∗
South	2354 (2187-2520)	5983 (5468-6498)	154.2∗	21.3 (19.77-22.79)	49.1 (44.84-53.28)	130.5∗
West	1061 (939-1183)	2783 (2250-3315)	162.3∗	15.3 (13.51-17.01)	36.5 (29.51-43.48)	139.2∗
Tenotomy						
Northeast	413 (315-512)	317 (201-433)	−23.3	7.6 (5.80-9.42)	5.7 (3.63-7.80)	−24.9
Midwest	592 (489-696)	711 (547-875)	20.1	9.0 (7.45-10.59)	10.6 (8.12-13.00)	17.1
South	509 (432-585)	618 (392-843)	21.3	4.6 (3.91-5.29)	5.1 (3.21-6.92)	10.0
West	283 (218-348)	811 (487-1135)	186.7∗	4.1 (3.13-5.01)	10.6 (6.39-14.89)	161.2∗

Incidence reported per 1,000,000.

Variables represented as estimates with 95% confidence intervals.

∗ Represents a statistically significant change.

### Trends in tenotomy and tenodesis in conjunction with RCR

**Figure 5:**
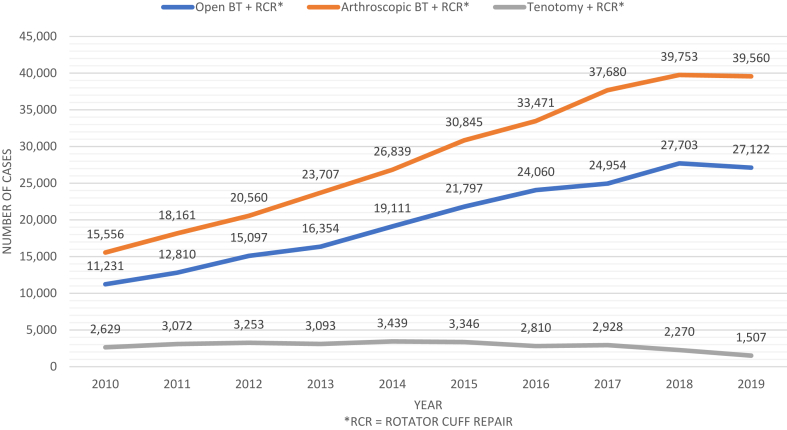
Case volumes: open vs. arthroscopic tenodesis with rotator cuff repair vs. biceps tenotomy with rotator cuff repair. *RCR*, rotator cuff repair; *BT*, biceps tenodesis.

A total of 209,951 and 299,917 open and arthroscopic biceps tenodesis procedures with concomitant RCR took place over the study period from 2010 to 2019, while only 30,704 biceps tenotomies with concomitant RCR occurred (Table I). Both open and arthroscopic tenodesis with concomitant RCR demonstrated upward trends, while tenotomy with concomitant RCR declined (Fig. 5). The incidence of open tenodesis with RCR increased by 126%, and the incidence of arthroscopic tenodesis with RCR increased by 138%, while the incidence of tenotomy with RCR decreased by 46% (Table VI). The distribution of the three procedures in the setting of RCR in 2010 was 38% open tenodesis, 53% arthroscopic tenodesis, and 9% tenotomy (Fig. 2). In 2019, LHBT procedures performed with RCR equaled 40% open tenodesis, 58% arthroscopic tenodesis, and 2% tenotomy.

**Table VI:** Volume and incidence estimates by year: open vs. arthroscopic biceps tenodesis with rotator cuff repair vs. biceps tenotomy with rotator cuff repair.

Y	Open tenodesis + RCR		Arthroscopic tenodesis + RCR		Tenotomy + RCR	
	Volume	Incidence	Volume	Incidence	Volume	Incidence
2010	11,231 (10,730-11,732)	36.9 (35.30-38.60)	15,556 (14,997-16,114)	51.2 (49.34-53.01)	2629 (2382-2875)	8.6 (7.84-9.46)
2011	12,810 (12,318-13,303)	41.8 (40.18-43.39)	18,161 (17,599-18,724)	59.2 (57.40-61.07)	3072 (2843-3301)	10.0 (9.27-10.77)
2012	15,097 (14,595-15,600)	48.8 (47.21-50.46)	20,560 (19,948-21,171)	66.5 (64.53-68.48)	3253 (2980-3526)	10.5 (9.64-11.41)
2013	16,354 (15,762-16,945)	52.5 (50.59-54.39)	23,707 (23,008-24,405)	76.1 (73.85-78.34)	3093 (2854-3332)	9.9 (9.16-10.70)
2014	19,111 (18,494-19,728)	60.8 (58.88-62.81)	26,839 (26,095-27,582)	85.4 (83.08-87.81)	3439 (3070-3807)	10.9 (9.77-12.12)
2015	21,797 (20,620-22,974)	68.9 (65.15-72.59)	30,845 (29,571-32,118)	97.5 (93.43-101.47)	3346 (2807-3885)	10.6 (8.87-12.27)
2016	24,060 (23,219-24,900)	75.5 (72.89-78.17)	33,471 (32,506-34,436)	105.1 (102.04-108.10)	2810 (2502-3118)	8.8 (7.85-9.79)
2017	24,954 (24,031-25,878)	77.7 (74.86-80.62)	37,680 (36,520-38,840)	117.4 (113.77-120.99)	2928 (2558-3298)	9.1 (7.97-10.27)
2018	27,703 (26,689-28,718)	85.8 (82.65-88.94)	39,753 (38,481-41,025)	123.1 (119.17-127.05)	2270 (1863-2677)	7.0 (5.77-8.29)
2019	27,122 (25,834-28,419)	83.5 (79.53-87.52)	39,560 (37,948-41,172)	121.8 (116.87-126.80)	1507 (1247-1767)	4.6 (3.84-5.44)
% change	141%	126%	154%	138%	−43%	−46%

*RCR*, rotator cuff repairs.

Incidence is reported per 1,000,000; % change represents the difference from 2010 to 2019.

Variables represented as estimates with 95% confidence intervals.

Trends were again investigated between the four age cohorts for LHBT management in the setting of RCR (Table VII). From 2010 to 2019, both open and arthroscopic tenodesis in the setting of RCR significantly increased, while tenotomy significantly decreased for all four groups. For the age <45 years cohort, the incidence of open and arthroscopic tenodesis increased by 117% and 123%, respectively. The distribution of LHBT procedures in the setting of RCR remained relatively constant in 2010 and 2019. In 2019, arthroscopic tenodesis remained the most common procedure for age <45 years in the setting of RCR (53%), and tenotomy in this cohort made up <1% (Fig. 3).

**Table VII:** Volume and incidence by age group: open vs. arthroscopic biceps tenodesis with rotator cuff repair vs. biceps tenotomy with rotator cuff repair.

	Volume			Incidence		
	2010	2019	% change	2010	2019	% change
Open tenodesis + RCR						
<45 yrs	1082 (961-1203)	2358 (2044-2672)	117.9∗	5.8 (5.15-6.45)	12.4 (10.75-14.06)	114.0∗
45-54 yrs	3653 (3434-3871)	7730 (7209-8252)	111.6∗	82.4 (77.52-87.38)	183.7 (171.34-196.14)	122.9∗
55-64 yrs	4856 (4603-5108)	12,365 (1,1715-13,016)	154.7∗	141.7 (134.28-149.03)	296.1 (280.57-311.70)	109.1∗
≥65 yrs	1641 (1284-1997)	4667 (3718-5617)	184.5∗	42.3 (33.14-51.54)	91.9 (73.21-110.60)	117.1∗
Arthroscopic tenodesis + RCR						
<45 yrs	1142 (1018-1265)	2640 (2326-2954)	131.2∗	6.10 (5.45-6.78)	13.9 (12.24-15.54)	127.9∗
45-54 yrs	4499 (4261-4737)	10,692 (10,085-11,299)	137.6∗	101.6 (96.18-106.93)	254.1 (239.70-268.55)	150.1∗
55-64 yrs	7435 (7127-7742)	18,979 (18,190-19,768)	155.3∗	216.9 (207.92-225.87)	454.5 (435.63-473.41)	109.5∗
≥65 yrs	2480 (2095-2866)	7249 (6011-8488)	192.3∗	64.0 (54.05-73.97)	142.8 (118.37-167.13)	123.1∗
Tenotomy + RCR						
<45 yrs	82 (51-113)	25 (0-50)	−70∗	0.4 (0.27-0.60)	0.1 (0.00-0.26)	−70.6∗
45-54 yrs	576 (491-661)	310 (208-412)	−46.1∗	13.0 (11.09-14.91)	7.4 (4.95-9.80)	−43.3∗
55-64 yrs	1198 (1076-1320)	783 (619-947)	−34.6∗	35.0 (31.39-38.52)	18.8 (14.83-22.69)	−46.3∗
≥65 yrs	773 (578-967)	389 (217-561)	−49.6∗	19.9 (14.92-24.96)	7.7 (4.27-11.05)	−61.6∗

*RCR*, rotator cuff repairs.

Incidence reported per 1,000,000.

Variables represented as estimates with 95% confidence intervals.

∗ Represents a statistically significant change.

The age >65 years cohort again underwent larger changes in procedure distribution over the study period (Fig. 3). In 2010, arthroscopic tenodesis made up 51% of LHBT procedures with concomitant RCR, and tenotomy accounted for 16%. Over the study period, open and arthroscopic tenodesis both significantly increased, while tenotomy significantly decreased. By 2019, the distribution of LHBT procedures in the setting of RCR became 59% arthroscopic tenodesis, 38% open tenodesis, and only 3% tenotomy.

When comparing trends in LHBT procedures with concomitant RCR between genders, both males and females had significant increases in open and arthroscopic tenodesis and significant decreases in tenotomy (Table VIII). In the setting of RCR, arthroscopic tenodesis remained the most common procedure for both males and females. Regarding the distribution of procedures, arthroscopic tenodesis increased in proportion while tenotomy decreased, with a greater decline seen in females (−10%) relative to males (−5%) (Fig. 4).

**Table VIII:** Volume and incidence by gender: open vs. arthroscopic biceps tenodesis with rotator cuff repair vs. biceps tenotomy with rotator cuff repair.

	Volume			Incidence		
	2010	2019	% change	2010	2019	% change
Open tenodesis + RCR						
Male	8012 (7577-8446)	18,957 (17,811-20,103)	136.6∗	53.6 (50.72-56.53)	118.6 (111.40-125.74)	121.1∗
Female	3219 (2967-3472)	8164 (7551-8778)	153.6∗	20.8 (19.19-22.46)	49.5 (45.82-53.26)	137.8∗
Arthroscopic tenodesis + RCR						
Male	10,160 (9701-10,619)	24,450 (23,188-25,711)	140.6∗	68.0 (64.94-71.08)	152.9 (145.03-160.81)	124.9∗
Female	5396 (5073-5718)	15,111 (14,098-16,123)	180.1∗	34.9 (32.82-36.99)	91.7 (85.54-97.83)	162.7∗
Tenotomy + RCR						
Male	1388 (1212-1565)	712 (526-897)	−48.7∗	9.3 (8.11-10.47)	4.5 (3.29-5.61)	−52.1∗
Female	1240 (1067-1413)	796 (613-978)	−35.8∗	8.0 (6.91-9.14)	4.8 (3.72-5.93)	−39.8∗

*RCR*, rotator cuff repairs.

Incidence reported per 1,000,000.

Variables represented as estimates with 95% confidence intervals.

∗ Represents a statistically significant change.

The four geographic regions followed similar trends with open and arthroscopic tenodesis in the setting of RCR, with significant increases among all four regions while tenotomy significantly decreased (Table IX). Furthermore, arthroscopic tenodesis remained the most common procedure in the setting of RCR in all four regions.

**Table IX:** Volume and incidence by region: open vs. arthroscopic biceps tenodesis with rotator cuff repair vs. biceps tenotomy with rotator cuff repair.

	Volume			Incidence		
	2010	2019	% change	2010	2019	% change
Open tenodesis + RCR						
Northeast	2198 (1964-2433)	3560 (3033-4088)	62.0∗	40.5 (36.18-44.82)	64.2 (54.70-73.72)	58.6∗
Midwest	3667 (3307-4027)	7328 (6796-7860)	99.8∗	55.8 (50.35-61.32)	108.9 (100.96-116.76)	94.9∗
South	3102 (2911-3293)	9539 (8791-1,0288)	207.5∗	28.0 (26.32-29.77)	78.2 (72.08-84.35)	178.9∗
West	2225 (2046-2404)	6694 (5936-7452)	200.9∗	32.0 (29.44-34.58)	87.8 (77.84-97.72)	174.2∗
Arthroscopic tenodesis + RCR						
Northeast	3582 (3271-3892)	5103 (4445-5762)	42.5∗	66.0 (60.26-71.69)	92.0 (80.16-103.93)	39.5∗
Midwest	4327 (4327-3977)	10,268 (9650-1,0885)	137.3∗	65.9 (60.56-71.22)	152.5 (143.34-161.69)	131.5∗
South	5242 (4992-5491)	16,593 (1,5542-1,7644)	216.5∗	47.4 (45.14-49.65)	136.1 (127.44-144.67)	187∗
West	2335 (2149-2520)	7596 (6757-8436)	225.3∗	33.6 (30.92-36.25)	99.6 (88.60-110.62)	196.5∗
Tenotomy + RCR						
Northeast	635 (502-768)	251 (139-363)	−60.5∗	11.7 (9.25-14.14)	4.5 (2.51-6.55)	−61.3∗
Midwest	904 (732-1077)	497 (345-648)	−45∗	13.8 (11.14-16.39)	7.4 (5.13-9.62)	−46.4∗
South	777 (681-873)	505 (362-648)	−35∗	7.0 (6.16-7.89)	4.1 (2.97-5.31)	−41.1∗
West	301 (236-366)	254 (145-364)	−15.6	4.3 (3.39- 5.27)	3.3 (1.91-4.77)	−23.1

*RCR*, rotator cuff repairs.

Incidence reported per 1,000,000.

Variables represented as estimates with 95% confidence intervals.

∗ Represents a statistically significant change.

## Discussion

Pathology involving the LHBT is a common pain generator of the shoulder, often experienced in conjunction with rotator cuff tears. After failure of conservative treatments, LHBT tenotomy or tenodesis are reliable and reproducible options with comparable outcomes.^1^^,^^2^^,^^17^^,^^20^^,^^28^ Although historically tenotomy and open subpectoral biceps tenodesis have been very successful, arthroscopic innovations over recent decades have seen arthroscopic LHBT tenodesis emerge as a viable alternative. Recent years have seen a tremendous number of comparison studies on LHBT tenotomy as well as open and arthroscopic tenodesis.^1^^,^^13^^,^^15^^,^^17^^,^^20^ With no proven gold standard technique^1^^,^^2^^,^^28^ or recent incidence studies evaluating the impact of these high-level comparisons, the purpose of this study was to evaluate surgical trends in the management of symptomatic LHBT in the United States, both as an isolated procedure and in the setting of RCR.

For isolated surgical management of a symptomatic LHBT, it appears that open subpectoral tenodesis remains the most common procedure in the United States over the last decade. From 2010 to 2019, the incidence of all isolated biceps procedures in the United States grew substantially, with open tenodesis growing by 180% and arthroscopic tenodesis increasing by 101%. By 2019, open tenodesis made up 59% of all isolated LHBT procedures, arthroscopic 35%, and tenotomy only 6%.

This observed increase in isolated tenodesis procedures, both open and arthroscopic, is in-line with trends observed from previous decades.^21^^,^^23^^,^^24^ Saltzman and colleagues not only noted increasing incidence of tenodesis procedures for isolated LHBT pathology from 2011 to 2014, but they also found open tenodesis to be more common than arthroscopic tenodesis as an isolated procedure.^21^ Despite the benefits of tenotomy including ease, shorter operative time, and cost,^27^ U.S. surgeons are clearly favoring tenodesis in recent years. Although it is hard to delineate the cause for this trend, the prevention of a “Popeye deformity” could likely be one of the driving factors.^12^^,^^25^^,^^28^ Patient preference could also play a role, as the idea of reinserting the tendon sounds more appealing than simply cutting the tendon. Finally, reimbursements in the setting of clinical equipoise may be a factor. According to the AAPC (formerly American Academy of Professional Coders), the relative value unit (RVU) for a tenotomy (CPT 23405) is 8.54, which is 16% less than an open tenodesis (CPT 23430) at 10.17 RVUs, and 35% less than arthroscopic tenodesis (CPT 29828) at 13.16 RVUs. Furthermore, arthroscopic tenotomy is often bundled into shoulder débridement codes such as 29822 and 29823. As a result of no true CPT code being available for arthroscopic tenotomy, it is difficult to determine the true number of these procedures from database studies such as ours.

When a concomitant RCR is performed, the gap between tenodesis and tenotomy grows even further. The incidences of open and arthroscopic tenodesis in the United States rose by over 100% in the setting of RCR. Contrary to the isolated procedure data, however, the incidence of tenotomy in the United States significantly decreased (−46%). By 2019, arthroscopic tenodesis accounted for 58% of LHBT procedures with concomitant RCR, followed by open tenodesis with 40%, and tenotomy only 2%. This pattern of large growth for tenodesis procedures and substantial decline of tenotomy with RCR remained consistent throughout subgroup analyses of gender, age, and region.

Prior work investigating surgical management of the LHBT in the setting of RCR has yielded mixed results. Our findings represent a shift from an earlier review by Jensen et al of Medicare data from 2005 to 2011, which demonstrated an increasing incidence of both tenotomy and tenodesis in the setting of RCR.^16^ Alternatively, our findings are supported by a subsequent study from 2011 to 2014 demonstrating higher rates of arthroscopic tenodesis compared to open in the setting of RCR and labral tears.^21^ Most recently, Cvetanovich et al reviewed the American Board of Orthopaedic Surgery database from 2012 to 2017, focusing on tenodesis in the setting of RCR, finding almost equal rates of open (52%) and arthroscopic (48%) biceps tenodesis.^7^

With our results demonstrating clear trends in the United States toward tenodesis in all scenarios, an interesting comparison is open vs. arthroscopic tenodesis. Our study found that open tenodesis remained the preference of U.S. surgeons over the arthroscopic technique as an isolated procedure, with open tenodesis making up 59% of procedures compared to arthroscopic being 35%. In the setting of RCR, however, the ratio of incidences flipped, with arthroscopic leading open tenodesis by 58% to 40% by 2019. This pattern is similar to the findings of Cvetanovich et al in their 2012 to 2017 American Board of Orthopaedic Surgery database review, in which they noted almost a 3:1 ratio of open to arthroscopic tenodesis as isolated procedures and a reduction to a 1:1 ratio in the setting of RCR.^7^

The trend toward arthroscopic tenodesis in the setting of RCR in the United States is likely multifactorial. Superior functional outcomes for arthroscopic tenodesis, however, have not been demonstrated.^10^^,^^11^^,^^13^ Infection may be a driving factor, as open tenodesis has been associated with a higher risk of surgical site infection.^1^^,^^13^^,^^14^ By utilizing an arthroscopic approach in conjunction with RCR, surgeons may perform tenodesis through existing portals, often needing only one additional small portal site for the tenodesis.^15^^,^^18^ Cosmesis can also be considered, as open tenodesis requires an additional incision, although proponents of the open technique argue that the incision is relatively inconspicuous for most patients. Additional factors to consider include surgeon training biases, skill, and time. Studies have produced mixed results on time differences between open and arthroscopic tenodesis,^9^^,^^26^ as operative time for an arthroscopic procedure can vary greatly based on surgeon training and confidence with the procedure. As surgeons performing RCR over the last decade are more likely to be fellowship trained and facile with arthroscopic techniques, it is not surprising that the trend is towards arthroscopic tenodesis.

Additional critical considerations for open vs. arthroscopic tenodesis are again cost and reimbursement. DeFroda et al found that open biceps tenodesis in the setting of RCR was associated with a cost (calculated from total charges) approximately $5000 lower than arthroscopic biceps tenodesis.^9^ It is postulated that this difference may be due to lower implant costs, fewer disposable instruments, and fewer surgical trays required with an open tenodesis.^9^ When looking at reimbursements, arthroscopic tenodesis (CPT 29828) is almost 30% higher than open tenodesis (23430): 13.16 RVUs compared to 10.17 RVUs.

Finally, stratifying by age allows for a unique assessment of procedural trends for LHBT management; specifically, cohorts at either end of the spectrum presented notable trends. Patients in the age <45 years cohort generally followed overall trends with the continued growth of open tenodesis in the isolated setting and the growth of arthroscopic tenodesis in the setting of RCR. Most notably, this age group saw tenotomy dwindle to a negligible proportion of LHBT procedures, making up <1% with concomitant RCR. Though a suspected trend in this age group, it confirms the current negative sentiment for tenotomy in younger patients and is in-line with findings from another trends study investigating LHBT procedures.^3^

Patients in the age ≥65 years cohort showed the most drastic changes in the growth of open tenodesis, both in isolation (1116%) and with RCR (185%). Those ≥65 years also experienced large growth in arthroscopic tenodesis in isolation (186%) and with RCR (192%), while also experiencing a substantial decline in tenotomy in the setting of RCR (−50%). Despite historic sentiment that low physical demands and fewer cosmesis concerns in this age cohort allow for tenotomy, it is clear this has changed. A combination of different factors is likely at play in relation to the decline in tenotomy, including decreased positive sentiment from surgeons towards tenotomy,^6^ along with better patient-reported outcomes and patient preference in the setting of tenodesis.^2^

There are of course some limitations to our study. By using a national insurance claims database, there are limitations inherent to large-scale databases and the generalizability of the data. Attempts were made to improve the generalizability by utilizing discharge weights when calculating the national procedural volume estimates. Discharge weights were provided by the database. Additionally, this study used CPT codes for open tenotomy, open tenodesis, and arthroscopic tenodesis. A specific code for arthroscopic tenotomy does not exist, and the procedure is often bundled into codes for arthroscopic shoulder débridement. Our results, therefore, likely underestimate the volume and incidence of arthroscopic tenotomy. The estimated volumes of open and arthroscopic tenodesis, however, are independent of this issue, and the documented trends remain valid. In addition, there is little data available through the IBM MarketScan database that would allow us to elucidate additional factors that might be causative of the trends seen in this study, including patient occupation and physical activity.

## Conclusion

This study documents trends associated with surgical management of a symptomatic LHBT, both as an isolated procedure and in the setting of RCR. The LHBT tenodesis procedure continues to be the preferred technique over a tenotomy alone and in the setting of a concomitant RCR. Open subpectoral tenodesis is the most common procedure for isolated LHBT pathology, while arthroscopic tenodesis has emerged as the preferred technique for concomitant RCR. Significant growth in arthroscopic and open tenodesis was noted in patients older than 65, while arthroscopic tenodesis volumes in patients younger than 45 were behind that of other age cohorts. Further investigation is needed to understand the factors driving these trends. This study can help serve as a foundation for future research to establish an evidence-based algorithm that can investigate factors related to indications, cost, and outcomes.

## Disclaimers:

Funding: No funding was disclosed by the authors.

Conflicts of interest: Michael Gottschalk is an associate editor for *JHS*; has received institutional research support from 10.13039/100008894Stryker, Konica Minolta, 10.13039/100007307Arthrex, and 10.13039/100014388Acumed. Eric Wagner is an associate editor at JHS GO and JAAOS; is a consultant for Stryker, Smith and Nephew, Depuy Synthes, and Acumed, and received institutional research support from Konica Minolta. The other authors, their immediate families, and any research foundation with which they are affiliated have not received any financial payments or other benefits from any commercial entity related to the subject of this article.
